# Transcriptomic Analysis of Rat Macrophages

**DOI:** 10.3389/fimmu.2020.594594

**Published:** 2021-02-01

**Authors:** Clare Pridans, Katharine M. Irvine, Gemma M. Davis, Lucas Lefevre, Stephen J. Bush, David A. Hume

**Affiliations:** ^1^Centre for Inflammation Research, University of Edinburgh Centre for Inflammation Research, Edinburgh, United Kingdom; ^2^Simons Initiative for the Developing Brain, University of Edinburgh, Edinburgh, United Kingdom; ^3^Mater Research Institute Mater Research Institute – University of Queensland, Brisbane, QLD, Australia; ^4^Faculty of Life Sciences, University of Manchester, Manchester, United Kingdom; ^5^UK Dementia Research Institute, University of Edinburgh, Edinburgh, United Kingdom; ^6^Nuffield Department of Clinical Medicine, University of Oxford, Headington, United Kingdom

**Keywords:** macrophage, colony-stimulating factor 1 receptor, rat, Kupffer cell, lipopolysaccharide

## Abstract

The laboratory rat is widely used as a model for human diseases. Many of these diseases involve monocytes and tissue macrophages in different states of activation. Whilst methods for *in vitro* differentiation of mouse macrophages from embryonic stem cells (ESC) and bone marrow (BM) are well established, these are lacking for the rat. The gene expression profiles of rat macrophages have also not been characterised to the same extent as mouse. We have established the methodology for production of rat ESC-derived macrophages and compared their gene expression profiles to macrophages obtained from the lung and peritoneal cavity and those differentiated from BM and blood monocytes. We determined the gene signature of Kupffer cells in the liver using rats deficient in macrophage colony stimulating factor receptor (CSF1R). We also examined the response of BM-derived macrophages to lipopolysaccharide (LPS). The results indicate that many, but not all, tissue-specific adaptations observed in mice are conserved in the rat. Importantly, we show that unlike mice, rat macrophages express the CSF1R ligand, colony stimulating factor 1 (CSF1).

## Introduction

The importance of the laboratory rat as a model for many human diseases, including cardiovascular, neurological, cancer, diabetes, respiratory and inflammatory disease has been widely-recognised [reviewed in (1, 2)]. The availability of whole genome sequences of multiple rat strains with well-characterised genetic disease susceptibility revealed evidence of selective sweeps associated with breeding for the trait, in many cases overlapping human disease susceptibility loci (3). Many of these diseases involve cells of the mononuclear phagocyte system (monocytes, tissue macrophages) in different states of activation as effectors. The underlying genetic susceptibility to disease has therefore been associated with differences in regulation or function of macrophage-expressed genes. For example, Maratou et al. (4) identified candidate genes underlying the differential sensitivity of rat strains to nephrotoxic glomerulonephritis by comparing the transcriptomes of their isolated macrophages, with and without stimulation with lipopolysaccharide (LPS).

Despite the extensive use of the rat as an experimental model, the gene expression profiles of rat macrophages have not been characterised to the same extent as mouse. Macrophages are a significant cellular component of all major organs and adapt in each site to perform specific functions. In mice, tissue-specific macrophage adaptation is associated with unique transcriptional profiles and expression of specific markers (5). Local adaptation is, in turn, driven by unique transcription factors; *Sall1* in microglia, *Gata6* in the peritoneum, *Nr1h3* in the marginal zone of spleen and in liver, *Spic* in the splenic red pulp, *Pparg* in alveolar macrophages, *Ahr* in Langerhans cells and *Batf3* in classical dendritic cells (6) (7–13).

Macrophage proliferation and differentiation is controlled by signals from the macrophage colony-stimulating factor receptor (CSF1R) in response to two alternative ligands, macrophage colony-stimulating factor (CSF1) or interleukin 34 (IL34). Macrophages generated *in vitro* from either monocytes, or bone marrow (BM) progenitors, by cultivation in CSF1, have been widely-used as models for the study of macrophage biology in multiple species (14–20). Monocyte-derived macrophages have not commonly been studied in mice, but can be accessed more readily in rats. Macrophages can also be generated from mouse embryonic stem cells (ESC), or induced pluripotent cells, through the intermediate of embryoid bodies, providing an avenue to analysis of macrophage-specific gene function without the necessity of producing live animals (21–25). The generation of ESC in rats was more challenging but is now routine and we and others have generated knockouts in rat ESC by homologous recombination [reviewed in (26)]. Accordingly, the same approaches to functional genome annotation through the use of ESC-derived macrophages are potentially available in rats.

We have used rat ESC to generate a knockout of the *Csf1r* locus. Whereas *Csf1r-*deficient mice have a severe phenotype, with few animals surviving to weaning, the rat knockout is viable as an adult and lacks many of the pleiotropic phenotypes seen in mice (27). The skeletal phenotype of *Csf1r*^-/-^ rats closely resembled homozygous recessive mutations in *CSF1R* in humans [reviewed in (28)]. A natural mutation in the *Csf1* locus in rats (*tl/tl*) also has a distinct phenotype to the CSF1-deficient op/op mouse (29). To dissect species-specific differences and to set the scene for more extensive use of the rat model, we decided to establish the methodology for production of rat ESC-derived macrophages and to compare their gene expression profiles to BM and monocyte-derived cells, and to various tissue macrophage populations. The results indicate that many, but not all, tissue-specific adaptations observed in mice are conserved in the rat.

## Materials and Methods

### Animals

Male Dark Agouti rats (8–10 weeks) were purchased from Charles River Laboratories (UK). *Csf1r* deficient rats and littermate controls were described previously (27). Approval was obtained from The Roslin Institute’s and The University of Edinburgh’s Protocols and Ethics Committees. The experiments were carried out under the authority of a UK Home Office Project Licence under the regulations of the Animals (Scientific Procedures) Act 1986. Animals were euthanized with rising concentrations of carbon dioxide.

### Alveolar and Peritoneal Macrophage Isolation

Alveolar (AM) and peritoneal macrophages (PM) were isolated as previously described for mice (30) and cultured in DMEM containing 10^4^ U/mL (100 ng/mL) recombinant human CSF1 (rhCSF1, a gift from Chiron, Emeryville, CA) as per (31). Following isolation, PM were allowed to adhere to non-tissue culture treated plastic (Sterilin) for 2 h before removal of non-adherent cells. Both AM and PM were then cultured overnight (37°C, 5% CO_2_) in rhCSF1 for further removal of non-adherent cells prior to flow cytometry analysis, RNA isolation and phagocytosis assays.

### Bone Marrow and Monocyte Derived Macrophages

Bone marrow cells were isolated from femurs as described (30) and cultured in DMEM containing rhCSF1 at a density of 2 × 10^5^ cells/cm^2^ on Sterilin plates. For isolation of peripheral blood mononuclear cells (PBMC), blood was collected into EDTA vacutainers (3S Health Care) *via* cardiac puncture. Blood was diluted 1:2 with PBS, layered on an equal volume of lymphoprep (Axis Shield) and centrifuged for 15 min, 1200 × *g* at RT with no brake. The PBMC layer was removed and topped up to 50 mL with media. After centrifugation at 400 X *g* for 5 min the pellet was resuspended in RBC Lysis buffer (BioLegend) according to instructions. After another wash step cells were resuspended in media containing rhCSF1 and seeded at a density of 1 × 10^5^ cells/cm^2^ on Sterilin plates. Fresh media was added on day 4 and cells used for flow cytometry analysis, RNA isolation and phagocytosis assays on day 7.

### Embryonic Stem Cell Culture

Embryonic stem cells (ESC) from Dark Agouti rats were derived as described in (32) and were obtained from Tom Burdon, The Roslin Institute, University of Edinburgh, UK. ESC were cultured on gamma-irradiated (5Gy) DR4 mouse embryonic feeder cells (Cambridge Stem Cell Institute, UK) in 2i media containing 10^3^ U/mL ESGRO Leukemia Inhibitory Factor (Millipore) as described in (32). DR4 cells were cultured on tissue culture plastic coated with 0.1% gelatin and inhibitors were custom-synthesized by the Division of Signal Transduction Therapy, University of Dundee, UK. Colonies were passaged every 2 days using TVP (0.025% trypsin, 1% chicken serum and 1 mM EDTA) and plated at a density of 5.5 x 10^4^ cells/cm^2^.

### Differentiation of Embryonic Stem Cells

One confluent well of a 6-well plate was used for differentiation of ESC into macrophages. On day 0 cells were passaged with TVP and placed in 5 wells of a 25-well plate (Sterilin) in 2i media. On day 2 the media was replaced with feeder media for embryoid body (EB) formation; GMEM containing 10% FCS (GE Healthcare), 2 mM GlutaMAX, 1 mM sodium pyruvate, 1X MEM-NEAA, and 0.1 mM 2-ME (Invitrogen). Media was replaced by transferring the cells to a 15 mL tube and allowing them to settle for 15 min at RT before aspirating spent media. EB were transferred to 9 cm petri dishes (Sterlin) on Day 7 in feeder media containing 5x10^3^ U/mL rhCSF1 and 10 ng/mL rat IL3β (Peprotech). Media was replaced or topped up every other day until day 18 (or when the first macrophages were observed to adhere to the petri dish). At this point the media was replaced and contained only rhCSF1 (10^4^ U/mL). The media was replaced regularly to remove debris. Macrophages were cultured for a further 7 days in rhCSF1 prior to flow cytometry analysis, RNA isolation and phagocytosis assays.

### RNA Isolation and Microarray

RNA was isolated using TRIzol (Life Technologies) according to instructions except 10% more chloroform was used during phase separation. Genomic DNA was removed with RNase-Free DNase followed by purification using RNeasy MinElute Cleanup Kit (QIAGEN, both according to instructions). RNA integrity and quality were assessed using the RNA ScreenTape Kit on the Agilent 2200 TapeStation. Samples with a RNA integrity number greater than 7 were used for microarray. Microarray was performed by Edinburgh Genomics (Edinburgh, UK) using Affymetrix Rat Gene 2.1 ST Array plates, and Expression Console 1.4.1.46 was used for quality control following amplification.

### Flow Cytometry Analysis of CD68 Expression

CD68 expression was used to assess the purity of cell populations prior to array analysis. Cells were harvested using a cell scraper and washed with PBS containing 1% bovine serum albumin (BSA). Cells were permeabilised using Leucoperm (AbD Serotec) according to instructions and incubated with mouse anti-rat CD68 (AbD Serotec, clone ED1, 1:200) followed by goat anti-mouse IgG APC (BioLegend, 1:400) and analysed on a FACSCalibur (BD). Data was analysed using FlowJo (Tree Star). Quadrants were set using the mouse IgG1 isotype control (AbD Serotec, clone F8-11-13).

### Phagocytosis Assays

Phagocytosis assays were performed as described (14) using Zymosan A BioParticles labelled with fluorescein (Invitrogen). Following fixation, cells were mounted with coverslips using ProLong Gold antifade reagent containing DAPI (Invitrogen).

### Microscopy

Cells were imaged using a LSM710 confocal microscope (Zeiss) or an Axiovert inverted microscope (Zeiss).

### Microarray Analysis – Rat Macrophage Gene Expression

The Affymetrix Transcriptome Analysis Console was used for hierarchical clustering and pairwise comparisons (**Figures 3A, B**). The arrays were then Robust Multi-array Average (RMA) normalised in R version 3.6.1. Graphia was used to generate gene-centred networks with a Pearson correlation of R = 0.85. Nodes where the maximum expression was <20 were removed. MCL clustering was used with an inflation value of 1.8 (**Figure 4**). Data was obtained from three adult wild-type rats on a DA background (AM, PM, MDM, and BMDM) and included two technical replicates per rat for BMDM. For ESDM, three technical replicates from the same ESC clone were used. Four array replicates were also included (BMDM, ESDM, PM, and MDM).

### Gene Set Enrichment Analysis

The enrichment of Molecular Signature (MSIG) database Gene Ontology sets, including BP, CC and MF (MSIG collection 5) in each macrophage population compared to the other 4 populations combined was analysed using Gene Set Enrichment Analysis (GSEA, Broad Institute) with phenotype permutation and default settings. For comparative analysis of rat and mouse macrophage signatures, we used GSEA to analyse the enrichment of 12 mouse macrophage signatures identified from cluster analysis of 466 RNA Sequencing datasets (cluster 7-microglia, 10-lung, 12-Kupffer cells, 13-CCR7^+^ DC, 15-monocytes, 21-peritoneum, 22-Lyve1^+^, 28-DC, 38-Intestinal macrophages, 43-Langerhans cells, 49-cDC1s, 3-mononuclear phagocytes) (5)in each rat macrophage population compared to all others.

### Microarray Analysis – Rat Kupffer Cell Gene Expression

The arrays were RMA normalised in R version 3.6.1. Using a Pearson correlation threshold cut-off of R = 0.85, nodes where the maximum expression was <10 and covariate of expression was <0.2 were removed. MCL clustering was used with an inflation value of 1.4. Data was obtained from wild-type, *Csf1r*^+/-^ and *Csf1r*^-/-^ rats (n = 4 per genotype).

### Microarray Analysis – LPS Time Course

The arrays were RMA normalised in R version 3.6.1. Using a Pearson correlation threshold cut-off of R = 0.85, nodes where the maximum expression was <100 were removed. MCL clustering was used with an inflation value of 1.8. Data was obtained from 3 adult wild-type rats on a DA background and included 2 technical replicates per rat. One array replicate was also included for 0 h LPS.

### Data Availability Statement

The full dataset has been uploaded to NCBI GEO (Accession Number GSE156188).

## Results

### Isolation, Generation and Characterisation of Rat Macrophage Populations *In Vitro*

**Figure 1:**
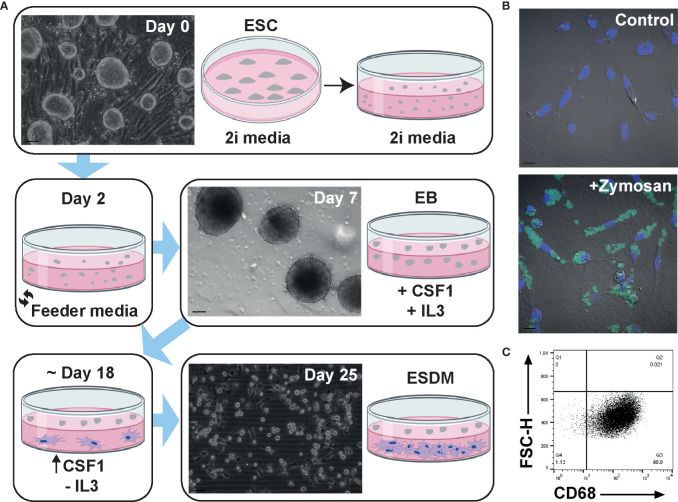
Generation and characterisation of rat embryonic stem cell derived macrophages. **(A)** Schematic diagram of rat embryonic stem cell (ESC)-derived macrophage differentiation. Confluent rat ESC are shown at day 0 (Clone DA5.2), embryoid bodies at day 7, and ESC-derived macrophages (ESDM) at day 25. Bars = 50, 100, and 50 µm, respectively. Images are representative of three repeat experiments, two replicates. **(B)** ESDM were cultured with or without (control) fluorescein labelled Zymosan A BioParticles. Images are representative of three repeat experiments, two replicates. Blue = nuclear DAPI staining. Bars = 10 µm. **(C)** Flow cytometry of permeabilized cells was used to assess purity of ESDM *via* CD68 expression. Quadrants were set using an isotype control. Dot plot is representative of three repeat experiments, two replicates.

The protocol for differentiating mouse ESC into macrophages has been well established (21, 33–35) but has not previously been applied to the rat. In brief, mouse embryoid bodies (EB) are plated on tissue culture (TC) grade plastic in the presence of both IL3 and CSF1. Macrophage progenitors are collected from the supernatant and plated on non-TC plastic in CSF1 alone. Embryonic stem cell-derived macrophages (ESDM) are then collected a week later (33). When this method was used for rat ESC no macrophages were produced (not shown). We therefore modified the protocol as follows. Rat ESC were grown on feeder layers in 2i media. When confluent (**Figure 1A**, day 0), cells were passaged and cultured in 2i media on non-TC plastic for the formation of EB and media was replaced on day 2 to remove LIF and the two inhibitors. Once EBs had formed (**Figure 1A**, day 7), IL3 and CSF1 were added to the culture media. When the first macrophages were observed to adhere to the non-TC culture dish (approximately day 18), the medium was replaced to contain CSF1 alone. Rat ESDM were then collected for analysis 7 days later (**Figure 1A**, day 25). The EB were cultured continuously in CSF1 and ESDM harvested as required, with cultures producing cells for at least 3 weeks. Rat ESDM were identified by their adherence to non-TC plastic, their characteristic morphology and their ability to phagocytose fluorescently-labelled Zymosan particles (**Figure 1B**). The monoclonal antibody ED1 recognises CD68 and is commonly used to identify rat macrophages (27, 36). We used flow cytometry to assess the purity of the ESDM prior to microarray analysis. Cells were routinely >95% CD68^+^ (**Figure 1C**).

**Figure 2:**
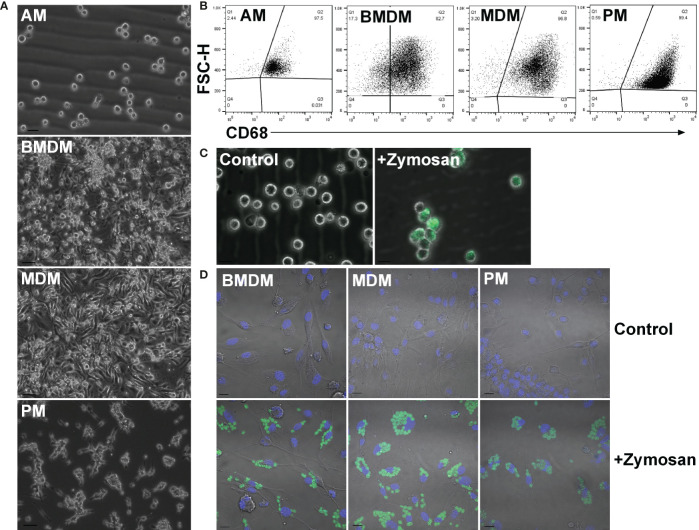
Adult rat macrophage populations. **(A)** Macrophages were isolated from adult male Dark Agouti rats. Images are representative of cells isolated from 10 rats. Bars = 20 µm (AM) or 50 µm (BMDM, MDM, and PM). **(B)** Permeabilized macrophages were analysed by flow cytometry to assess purity *via* CD68 expression. Quadrants were set with isotype controls. Dot plots are representative of cells isolated from three rats. **(C)** Alveolar macrophages were cultured with or without (control) fluorescein labelled Zymosan A BioParticles and imaged with a Zeiss AxioVert. Images are representative of cells isolated from three rats. Bar = 50 µm. **(D)** Macrophages were cultured with or without (control) fluorescein labelled Zymosan A BioParticles and imaged with a Zeiss LSM 710 confocal. Images are representative of cells isolated from three rats. Bar = 10 µm. AM = alveolar macrophages, BMDM = bone marrow derived macrophages, MDM = monocyte derived macrophages, PM, peritoneal macrophages.

To compare ESDM to more widely-studied populations and to assess tissue-specific adaptation, rat alveolar (AM), peritoneal (PM), BM-derived (BMDM) and monocyte-derived (MDM) macrophages were isolated and cultured as outlined in the Materials and Methods (**Figure 2A**). Because of the low yield of monocytes from mouse blood, the generation of MDM in mouse is not straightforward. By contrast, an average of 1.7 x 10^6^ peripheral blood mononuclear cells (PBMC) were isolated per mL of rat blood. To increase the purity of AM and PM *ex vivo*, cells were cultured overnight on non-tissue culture plastic to remove non-adherent (i.e. non-macrophage) cells. AM and PM were cultured in CSF1 to mitigate potential loss of viability as mouse macrophages require CSF1 for survival (37). The purity of populations was confirmed by flow cytometry staining for CD68 (**Figure 2B**). Phagocytic activity was confirmed by assaying the uptake of fluorescently labelled Zymosan particles (**Figures 2C, D**).

### Pairwise Comparison of Rat Macrophages

**Figure 3:**
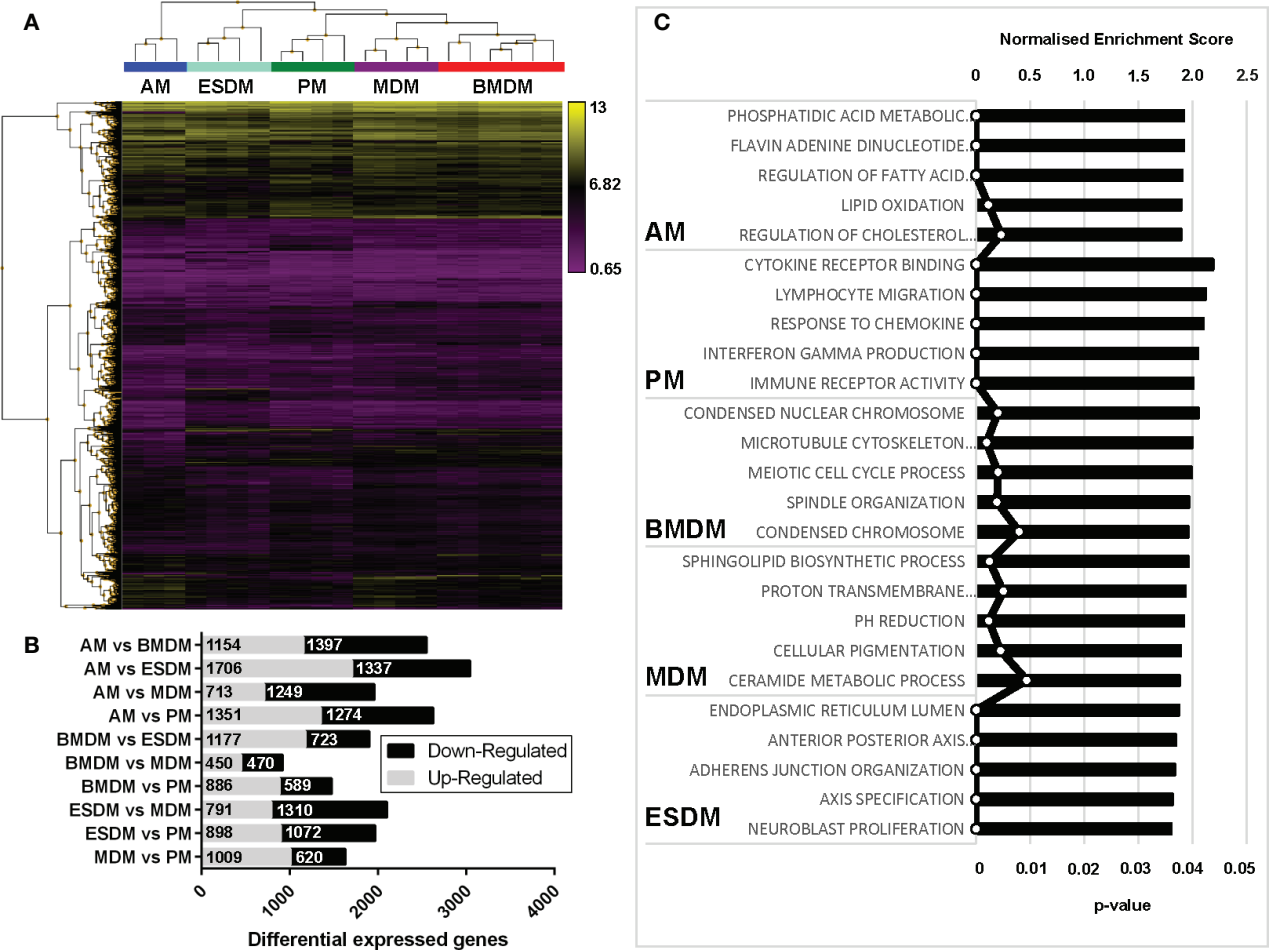
Comparison of rat macrophage gene expression *in vitro*. Microarray analysis was performed on *in vitro* cultured rat macrophages. **(A)** Hierarchical clustering of the top 5,000 differentially expressed genes sorted by false discovery rate (FDR; <0.009418). Three biological replicates were used for alveolar (AM), bone marrow-derived (BMDM), monocyte-derived (MDM) and peritoneal macrophages (PM), including technical replicates. Technical replicates were used ESC-derived macrophages (ESDM) obtained from a single ESC clone (DA5.2). **(B)** Graph shows the numbers of differential expressed genes for each of the rat macrophages. **(C)** Gene ontology-based gene sets that were enriched in each macrophage population compared to all the others were identified using Gene Set Enrichment Analysis.

To identify the similarities and differences among the primary and culture-derived macrophage populations we performed microarray analysis. A pairwise comparison was performed in the Affymetrix Transcriptome Analysis Console and **Figure 3A** shows hierarchical clustering of the top 5000 differentially expressed genes (DEGs). In our previous study, pig BMDM and MDM were largely indistinguishable (17). Consistent with this, rat BMDM and MDM were more similar to each other than to any of the other populations. Comparing their expression profiles using the Affymetrix Expression Console identified there were only 920 differentially expressed genes (**Figure 3B**). The complete gene lists and principle component analysis (PCA) plot are located in **Table S1** and **Figure S1a**, respectively. Alveolar macrophages are quite phenotypically different from other mouse tissue macrophage populations as they are dependent on GM-CSF, rather than CSF1 for survival (38). Indeed, rat AM clustered on their own compared to the other macrophage populations (**Figure 3A**).

We next identified gene ontologies that were enriched in each macrophage population compared to all the others using gene set enrichment analysis (GSEA) (**Figure 3C**). AM were significantly enriched for genes involved in fatty acid metabolism, consistent with their role in lung surfactant protein metabolism (39). PM DEG were enriched for cytokine, chemokine and immune receptor activity, perhaps reflecting their critical roles in peritoneal defence. BMDM were significantly enriched for cell cycle-related genes, indicating a higher level of ongoing proliferation compared to MDM and ESDM. The ESDM showed enrichment for developmental pathways such as anterior-posterior axis specification but we cannot eliminate some contamination with embryoid body derived material.

### Proliferation and CSF1 Signalling in Isolated Macrophages

**Figure 4:**
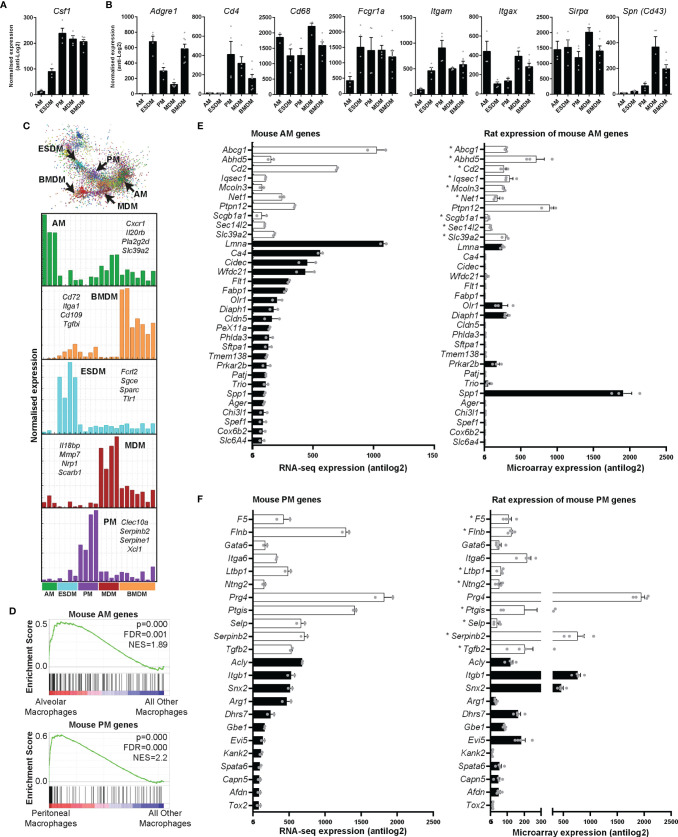
Expression of rat macrophage genes. Microarray analysis was performed on *in vitro* cultured rat macrophages. Data were RMA-normalised and expression levels (antilog2) examined. **(A)** Expression of *Csf1* in rat macrophages. **(B)** Expression of macrophage-specific genes encoding commonly-used surface markers including those for which there are no anti-rat antibodies. Graphs **(A**, **B)** show average + SEM. **(C)** The network graph generated by Graphia analysis in which genes are coloured by clusters of co-expression. Histograms show expression profiles of clusters that contained genes specific to each macrophage population. The genes listed encode cell surface proteins. AM = alveolar macrophages, BMDM = bone marrow derived macrophages, MDM = monocyte derived macrophages, PM = peritoneal macrophages, ESDM = embryonic stem cell derived macrophages. **(D)** Global enrichment of mouse macrophage AM and PM signature gene sets (5) in rat AM and PM identified by Gene Set Enrichment Analysis. Rat expression data are ranked according to differential expression in AM or PM compared to all other macrophage populations (indicated by red-blue bars), and the murine AM or PM genesets are mapped onto this profile (black bars) to determine enrichment score (green lines). NES = Normalised Enrichment Score. **(E**, **F)** The differentially expressed genes identified by Lavin and colleagues (44) were clustered using Graphia. The graphs show expression of genes identified in the mouse alveolar (AM) and peritoneal (PM) macrophage clusters. The genes represented by open bars were common to both mouse and rat AM clusters. Rat genes denoted with an asterisk (*) have provisional or model RefSeq status on the Rat Genome Database (rgd.mcw.edu). Graphs show mean + SEM.

All of the isolated macrophages expressed abundant *Csf1r* mRNA. Proliferating mammalian cells induce a suite of genes required to transit of S phase and mitosis (40). Although the levels were highest in BMDM, each of the macrophage populations expressed detectable *Mki67* and *Pcna* (which encode widely-used proliferation-associated nuclear proteins), cell cycle transcription regulators (*Foxm1*, *E2f*), cyclins and many other annotated cell cycle genes, indicating active proliferation in the presence of exogenous CSF1. Each population expressed similar levels of direct CSF1 target genes, the transcription factor *Ets2*, members of the *Jun* family and urokinase plasminogen activator (*Plau*) (41–43). Unlike mouse BMDM, which undergo apoptosis when exogenous CSF1 is removed (37); rat BMDM once differentiated do not require exogenous CSF1 for survival (not shown). Consistent with the existence of a basal autocrine loop, each of the macrophage populations (with the exception of AM) expressed abundant *Csf1* mRNA (**Figure 4A**).

### Macrophage-Specific Genes in the Rat

Antibodies against SIRPα (CD172a), CD11b/c, CD4, SPN (CD43), and intracellular CD68 are used to identify rat monocytes and macrophages *via* flow cytometry (27, 45). An AF647 conjugated CSF1-Fc fusion protein can also be used to identify CSF1R expressing cells in rats (27). We first analysed the expression of these markers in our macrophage populations. **Table S2** includes the RMA-normalised data. *Cd68* and *Sirpα* were highly expressed in all rat macrophages (**Figure 4B**). In mice, CD4 is commonly used to identify a subset of T cells. With the exception of subsets of macrophages in the wall of the gut (46), CD4 is not detected in mouse macrophages. In rats, the anti-CD4 antibody (W3/25 antigen) also detects peritoneal macrophages (47) and we have previously shown that CD4 is expressed by rat monocytes (27). In the isolated rat macrophages, *Cd4* was highly-expressed by BMDM, MDM and PM but just detectable in AM and ESDM. As in mice (5), *Itgam* (*Cd11b*) expression was highest in PM. *Itgax* (*Cd11c*) is still widely considered a dendritic cell (DC) marker in mice, despite clear expression by many tissue macrophage populations [reviewed in (48)]. *Itgax* is not expressed by mouse BMDM (Biogps.org), whereas in rats it was abundant in all the macrophage populations, and highest in AM, BMDM and MDM. The E-selectin ligand sialophorin (SPN) (CD43) is highly expressed on the non-classical monocyte population in blood and BM (equivalent to Ly6C^lo^ in mice) (45). The differentiation of these cells is dependent upon CSF1R signalling (28). *Spn* mRNA was highly-expressed in MDM and BMDM but much lower in PM and almost absent in AM (**Figure 4B**).

We also looked at expression of genes which encode surface markers commonly used to define and separate (sub)populations of mouse macrophages (5, 49) (as above). We recently characterised a monoclonal antibody against pig ADGRE1 (F4/80) and found that the antigen was highly-expressed by pig AM (50). *Adgre1* is part of a family of five related genes, encoding adhesion G protein coupled receptors, which are divergent across species in sequence and copy number (50). Like mice, rats have only 3 members of the family (*Adgre1*, *4*, and 5), whereas humans and other large animals have an additional 2 members (*ADGRE2* and *3*) which are also expressed in myeloid cells. *Adgre1* mRNA was most abundant in rat BMDM and ESDM, (**Figure 4B**). As in mice (6) there was almost no expression in AM. CD64 (encoded by *Fcgr1*) and the apoptotic cell receptor MERTK were proposed as markers to distinguish macrophages from DC in various mouse tissues (51, 52). *Fcgr1a* was highly-expressed by all the isolated rat macrophages, but somewhat lower in AM (**Figure 4B**).

To identify genes specific to individual macrophage populations, we clustered the array data using the network analysis tool Graphia (53). The gene-centred network generated by Graphia is based upon the Pearson correlation co-efficient between individual genes and requires no supervision other than the setting of a threshold R value. This is chosen at the inflexion point to maximise the number of nodes (genes) included in the network graph whilst minimising the number of edges (correlations). There were five clusters of genes in which the expression was clearly higher in one macrophage population compared to the others (**Figure 4C**). The list of genes associated with each cluster is within **Table S2** and **Figure 4C** shows the mean expression histograms for each cluster. We have listed a few genes encoding cell surface molecules that are specific for each macrophage population that may be useful for monoclonal antibody production in the future (**Figure 4C**).

For comparative analysis of rat and mouse macrophage signatures, we first analysed the enrichment of 12 mouse macrophage signatures identified from cluster analysis of 466 RNA Sequencing datasets, including microglia, lung, Kupffer cells, CCR7^+^ DC, monocytes, peritoneum, Lyve1^+^ macrophages, DC, intestinal macrophages, Langerhans cells, cDC1s, and a cluster common to all mononuclear phagocytes (5), in each rat macrophage population compared to all the others using Gene Set Enrichment Analysis. The mouse AM and PM signatures showed significant enrichment in the rat AM and PM populations, respectively (**Figure 4D**), whereas no significant enrichment was observed for rat ESDM, BMDM, or MDM. This indicates similarity between rat and mouse AM and PM populations at a global level. However, it was also apparent from this analysis that many mouse macrophage signature genes were more highly expressed in other macrophage populations than in the ‘signature population’ (**Figure 4D**).

We then examined individual genes by comparing expression between mouse AM and PM signatures identified by Lavin and colleagues (44) with our rat data. The differentially expressed genes they identified between mouse monocytes, microglia, and macrophages isolated from the liver, spleen, lung, peritoneal cavity and intestine were clustered in Graphia to obtain unique gene lists for each cell type (**Figure S2** and **Table S3**). The majority of genes identified as AM specific by Lavin et al. data were not RefSeq validated in the rat (**Figure S3**). There were 10 genes common to both mouse and rat AM clusters (*Abcg1*, *Abhd5*, *Cd2*, *Iqsec1*, *Mcoln3*, *Net1*, *Ptpn12*, *Scga1a1*, *Sec14l2* and *Slc39a2*) (open bars in **Figure 4E**). Of these genes, *Ptpn12* had the highest expression and was the only RefSeq validated gene in this group. **Figure 4E** shows expression of the common and validated genes. Only 5 of 22 genes identified in mouse AM showed expression in rats. These differences could arise from the isolation method. Lavin et al. digested the lungs to isolate AM, whereas in the rats the lungs were lavaged. Siglecf is commonly used as an AM marker in mice in flow cytometry analysis (54). The ortholog is *Siglec5* in rats which only has a provisional RefSeq status. In our data, *Siglec5* was most highly expressed in ESDM (**Table S2**). A higher percentage of mouse PM genes were expressed in the rat. **Figure 4F** shows the 11 genes that were common to both mouse and rat PM clusters (*F5*, *Flnb*, *Gata6*, *Itga6*, *Ltbp1*, *Ntng2*, *Prg4*, *Ptgis*, *Selp*, *Serpinb2*, and *Tgfb2*). It is worth noting the inclusion of *Gata6* in the rat PM cluster, as this gene has been previously shown to be down-regulated in the presence of CSF1 in mice (55). Ten of 12 RefSeq validated genes identified in mouse PM showed expression in rats.

### Inferred Gene Expression Profile of Rat Kupffer Cells

**Figure 5:**
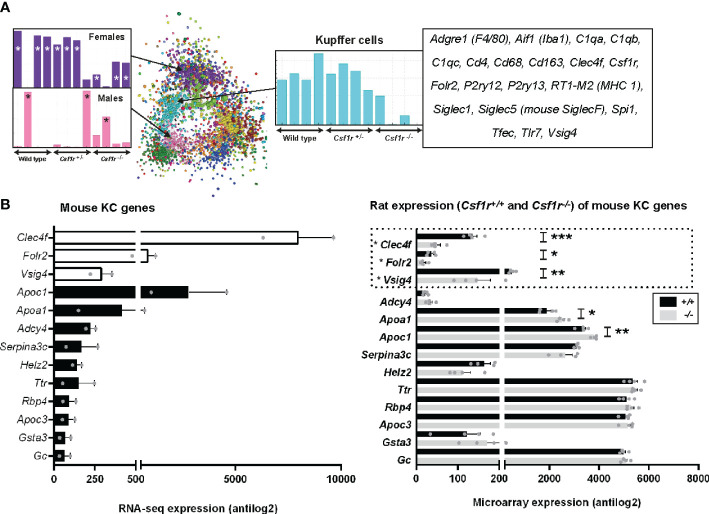
Inferred gene expression profile of rat Kupffer cells. **(A)** Microarray analysis was performed on adult livers obtained from wild type, *Csf1r*^+/-^ and *Csf1r*^-/-^ rats (n = 4 per genotype). Data were RMA-normalised and analysed using Graphia. **(A)** The network graph generated by the Graphia analysis. Genes are coloured by clusters of co-expression. Expression profiles of clusters that contained genes specific to Kupffer cells (cluster 4), females (cluster 3) and males (cluster 7) are shown. **(B)** The differentially expressed genes identified by Lavin and colleagues (44) were clustered using Graphia. The graphs show expression of genes identified in the mouse Kupffer cell (KC) cluster. The genes represented by open bars or within the dotted line were common to both mouse and rat KC clusters. Rat genes denoted with an asterisk (*) have provisional or model RefSeq status on the Rat Genome Database (rgd.mcw.edu). Graphs show mean + SEM. p = 0.0007 (*** *Clec4f*), 0.0414 (* *Folr2*), 0.0024 (** *Vsig4*), 0.0209 (* *Apoa1*), and 0.0091 (** *Apoc1*) *via* an unpaired t-test.

The resident macrophages of the liver (Kupffer cells, KC) are amongst the largest populations of macrophages in the body. There are several published methods for the isolation of rat KC, generally involving enzymatic perfusion of the liver (56–60). We previously inferred the gene expression profile of neonatal rat KC based upon the set of genes that was induced in the liver by treatment with CSF1 (19). The recent development and characterisation *Csf1r*-deficient rats permitted the analysis of brain (microglia) and spleen-specific macrophage gene expression profiles based upon the identification of transcripts that were selectively lost in the whole tissue gene expression profiles (27). To assess the gene expression profiles of adult rat KC *in situ* we analysed whole liver microarray data from *Csf1r*^+/+^, *Csf1r*^+/-^, and *Csf1r*^-/-^ rats using Graphia (53). *Csf1r*^-/-^ rats exhibit a reduction in CD68^+^ KC by immunohistochemical staining and are also monocyte deficient (27). The complete gene lists and principle component analysis (PCA) plot are located in **Table S4** and **Figure S1B**, respectively. Using a Pearson correlation threshold cut-off of R = 0.85 we identified a cluster of genes which included *Csf1r* (cluster 4, **Figure 5**). These genes were expressed in wild type and *Csf1r*^+/-^ rats, but substantially reduced in *Csf1r*-deficient rats (**Table S4**). The set of CSF1R-dependent genes reduced in the homozygotes includes *Cd163* (commonly used in KC isolation in rats), *Cd4*, *Cd68*, *Adgre1*, and *Aif1* (encoding the marker IBA1) and the extent of the reduction is consistent with the relative loss of KC observed in *Csf1r*^-/-^ rats by immunohistochemistry (27). This list also includes KC-specific genes identified in mouse and/or human, notably *Clec4f* and *P2ry13* (44, 61). However, the large majority of putative KC-specific transcripts identified by profiling isolated KC in mice (44) and humans (61) were not *Csf1r*-dependent in rat liver. Some, such as *Marco* and *Timd4*, were barely detected in total rat liver mRNA. Others such as *Mertk* and *Mrc1*, were expressed but not significantly reduced in the *Csf1r-*deficient rats (**Table S4**). Three of the rats in each group were female. We also identified two clusters that appeared sex-specific and affected by *Csf1r* mutation (**Figure 5**). cluster 3 includes genes encoding the transcription factors *Cux2* and *Tsx* and target genes (*Ascl1*, *Lifr*, *Prlr*) that were down-regulated in the female *Csf1r*^-/-^ livers. cluster 7 contained male-specific genes such as *Bcl6* and targets *Cyp2c11*, *Sult1e1* and *Hsd3b5* that were highly up-regulated compared to the female samples. These differences are likely related to impaired gonad development and infertility in these rats (27).

For comparative analysis of rat and mouse KC signatures, we compared genes in our rat cluster to data obtained from Lavin and colleagues (44) as described above for AM and PM. There were only 3 genes shared between the two datasets, *Clec4f*, *Folr2*, and *Vsig4* (open bars **Figure 5B**). Again, the vast majority of genes identified as KC specific in Lavin’s data were not RefSeq validated in the rat (**Figure S5**). Of the 45 KC genes in the mouse, 39 of these showed no difference in expression between wild-type and *Csf1r*^-/-^ rat livers, suggesting they are not rat KC markers (**Figure 5B** and **Figure S5**). Six genes were statistically different between the genotypes, however, their expression increased with a loss of *Csf1r*-dependent macrophages in rat liver.

### Transcriptional Response to LPS in Rat BMDM

**Figure 6:**
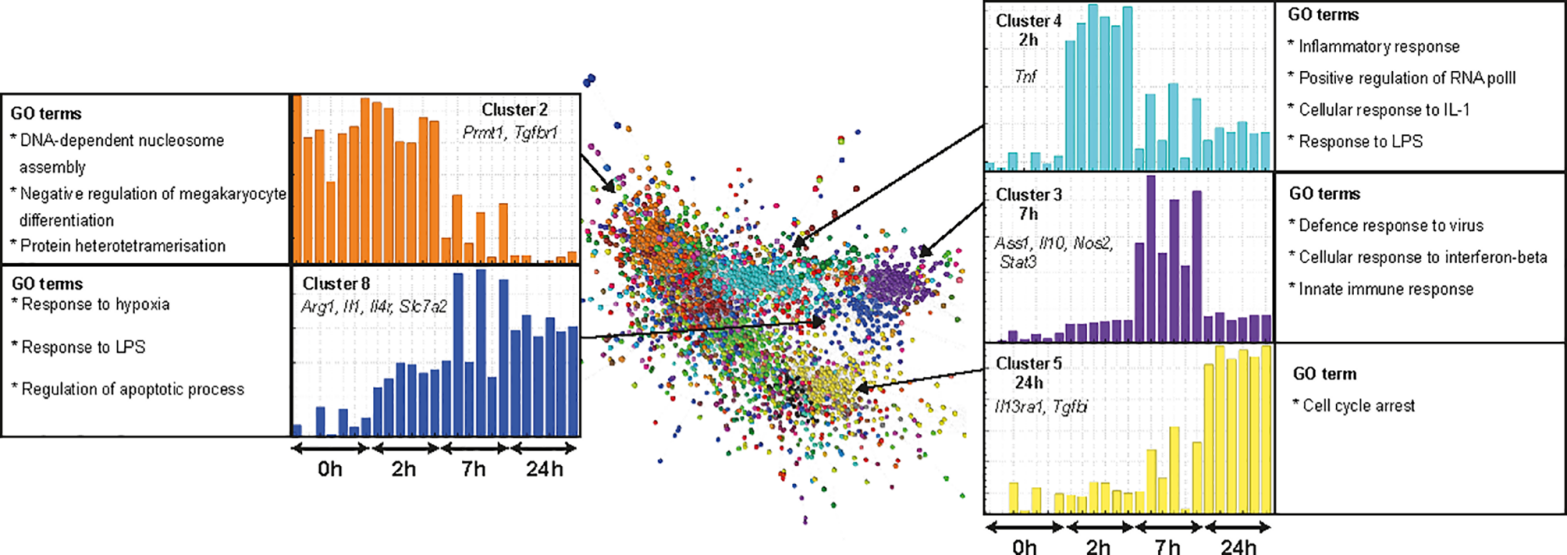
Transcriptional response of rat BMDM to LPS. Adult male rat bone marrow (BM) was differentiated into macrophages for 7 days in CSF1. RNA was isolated from BM-derived macrophages (BMDM) after culture with LPS (0, 2, 7, and 24 h) and microarray analysis was performed. Data were RMA-normalised and analysed using Graphia. The network graph generated by the Graphia analysis is shown. Genes are coloured by clusters of co-expression. Expression profiles of clusters that contained genes specific to each time point are shown. Data was obtained from three adult wild-type rats on a DA background and included two technical replicates per rat. One array replicate was also included for 0 h LPS.

Lipopolysaccharide (LPS) molecules are endotoxins that are found in the outer wall of gram-negative bacteria and have been widely used in mouse models of inflammation, both *in vitro* and *in vivo* [reviewed in (62)]. Binding of endotoxins to pattern recognition receptors such as toll-like receptor 4 (TLR4) results in the induction of genes required by macrophages to elicit an effective defence against pathogens. Several studies have compared the transcriptomic response of BMDM or MDM to LPS in humans, small and large ruminants, horses, pigs, rats and mice (20, 63–65). The rat data in that study was based on a single time point (7 h) but the response to LPS is a sequential cascade of transient gene expression (20, 63). To more accurately capture that profile, we cultured rat BMDM in LPS for 0, 2, 7, and 24 h. Microarray data was obtained from 3 rats and included technical replicates. Using Graphia (53) and a Pearson correlation threshold cut-off of R = 0.85 we identified five main gene clusters (**Figure 6**). Consistent with analysis in other species, two of these clusters (3 and 4) had an average profile that presented transient induction, peaking at 2 and 7 h, respectively. The complete gene lists and principle component analysis (PCA) plot are located in **Table S5** and **Figure S1C**, respectively and are discussed below.

## Discussion

The resident macrophages in every tissue adapt to the local environment to perform specific functions (66). The large majority of the current knowledge of macrophage differentiation and homeostasis is derived from studies of mice. However, there is increasing recognition that the insights derived from a single inbred mouse strain may not be generalisable to other mammalian species (67, 68). We have invested in the rat as an alternative model (19, 27, 45). Analysis of *Csf1r*^-/-^ rats revealed major differences compared to the equivalent mouse mutation, which may be related to the fundamental difference in macrophage expression of the growth factor gene, *Csf1*, confirmed here. In this study we have expanded the set of tools and macrophage expression data available for the rat. We have generated and characterised MDM and ESDM, and extended knowledge of tissue-specific macrophage adaptation from previous inferred expression profiles of rat microglia and splenic macrophages (27).

### Characterisation of ESDM and MDM

The original studies that generated mouse ESDM (25) recognised their potential for studying macrophage gene function, a potential that has been fulfilled in subsequent studies (21, 33–35, 49) and extended to human systems (69). The generation of pure macrophage populations from rat ESC was relatively straightforward and enabled repeated harvesting over several weeks. With the increasing ease of genetic manipulation in ESC using CRISPR-Cas9 this provides a convenient system for analysis of rat macrophage differentiation. Comparative analysis of the expression profiles of ESDM and BMDM revealed around 1500 differentially-expressed genes. None of the differences was absolute and few were obviously associated with macrophage function. For example, there was no significant difference in expression of toll-like receptors. The transcripts over-expressed by ESDM included *Sall1*, *Cx3cr1*, and *C1qa* genes enriched in microglia, whereas BMDM expressed higher *Cd4*, *Cd74*, and *Fcgr2b*. Based upon the rapid turnover and CSF1-dependence of intestinal macrophages, human MDM grown in CSF1 have been proposed as an *in vitro* model of intestinal macrophages. Compared to monocytes, they exhibit a damped response to stimuli such as LPS (63), with relatively lower pro-inflammatory (IL1) and higher anti-inflammatory cytokine production. As previously shown in the pig (17) rat MDM are very similar to BMDM; the differentially-expressed genes were not obviously enriched for any receptors, secretory products or effectors.

### The Response of Rat BMDM to LPS

The LPS time course for rat BMDM is directly comparable to data produced previously with the same cell culture conditions as mouse, pig and human (64, 65) and more recently RNA-seq data from a wider range of species (20). The rat BMDM resemble mouse, and differ from human, horse and pig, in that all of the genes required for arginine metabolism and the generation of nitric oxide (e.g., *Arg1*, *Ass1*, *Gch1*, *Nos2*, and *Slc7a2*), were induced by LPS, whereas genes involved in tryptophan metabolism (*Ido1*, *Kynu*, *Kmo*) were not. They differ from mouse BMDM in that many of the immediate early genes (*Dusp1*, *Egr*, *Fos*, *Ier*, *Jun*, and *Nr4a1*) that are rapidly induced by LPS in mice were expressed constitutively in rat BMDM and not further regulated. One such gene that has been the focus of studies in mice is *Acod1*, which diverts citrate from the TCA cycle to the production of itaconate, a proposed feedback regulator of inflammation [reviewed in (70)]. By contrast to mouse BMDM, in which *Acod1* was barely detectable and rapidly and massively induced by LPS (71), in the rat BMDM *Acod1* was abundant in unstimulated cells and induced only 3–5 fold. The temporal cascade of gene expression in rat BMDM was similar to mouse and human, in that a peak of transient gene expression at 2 h included a massive peak of expression of *Tnf* and key transcriptional regulators such as *Irf1*. This was followed by a second transient peak at 7 h that included transcription factors (e.g., *Stat1*, *Irf7*) numerous interferon response genes (e.g., *Cxcl10*, *Ifit1*, *Isg15*, *Mx1*, and *Oas1b*) and the feedback regulator *Il10*. A second difference between rat and mouse BMDM is that the LPS response in the rat was more sustained, with pro-inflammatory cytokines *Il1a*, *Il1b*, and *Il6* still maximally induced after 24 h. One explanation for the temporal difference between rat and mouse may relate to the difference in CSF1 expression. In mouse BMDM, CSF1 enhances the response to LPS whilst down-regulating the response to TLR9 agonists (72). Conversely, LPS activates a subset of genes in mouse BMDM by blocking CSF1 signalling (73). All of these genes (including *Tlr9*, *Ramp2*, and *Itm2b*) were expressed in rat BMDM but not induced by LPS.

### Alveolar and Peritoneal Macrophages

There are some overlaps, but major differences in both the macrophage-specific gene expression in mice and rats and the underlying transcriptional regulation. A detailed comparison of AM, PM, splenic and brain (microglia) macrophages was published by the ImmGen Consortium (52) and the comparison is extended in a recent meta-analysis of mouse macrophage RNA-seq data (5).

Comparative functional analyses of rat peritoneal and alveolar macrophages have a long history (74–76) and references therein). Consistent with those earlier studies, genes encoding proteins such as dipeptidyl aminopeptidase II (*Dpp7*), cathepsin D (*Ctsd*), and CD206 (*Mrc1*) were elevated >2-fold in AM compared to PM but were also highly-expressed in the culture-derived macrophages. Conversely, we confirmed strong selective expression of genes associated with fatty acid (arachidonate) metabolism in AM, including prostaglandin synthetic enzymes [*Ptgs1* and *Ptgs2*, previously shown at protein level (77)] and protein kinase C (*Prkca*) (78, 79). However, other genes associated with arachidonate metabolism had relatively low expression (*Alox5*, *Ptgis*, and *Pla2g16*).

We also confirm selective and very high expression of pre-protachykinin (*Ppt1*) (80) and identity N-acylethanolamine-hydrolyzing acid amidase (*Naaa*) as a unique rat AM marker (81). We confirm the early report (82) that rat AM have relatively low levels of Cd11b (*Itgam*) and class II MHC [*RT1-Ba/b*, *RT1-Db1*, and *RT1-DOa*)]. The set of AM-enriched genes contains several other candidate surface markers (**Table S2**). One with exceptionally high expression, that is almost absent from all the other macrophage populations, is the chemokine receptor, *Cxcr1*, the receptor for Cxcl8 (IL8). Although Cxcl8 is regarded as a neutrophil chemoattractant, when it was originally cloned in rats, *Cxcr1* mRNA was detected in rat lung and isolated macrophages (83). Neither rats nor mice have an annotated *Cxcl8* gene; binding studies identified Cxcl6 as the ligand of this receptor in rodents (84). The function of this receptor in rat AM is unclear.

In mice, resident peritoneal macrophages express *Gata6* (85) and so do rat PM. Rat PM express much higher levels of class II MHC than mouse. MHCII-expressing peritoneal macrophages in mice depend upon the transcription factor IRF4 (86), which was, indeed, strongly expressed in rat peritoneal macrophages. Gata6 was inferred to be a regulator of murine peritoneal macrophage survival, in part by regulating metabolism and expression of key enzymes such as aspartoacylase (*Aspa*) (87). However, the level of mRNA encoding this enzyme was actually >3-fold higher in rat AM than PM.

Amongst the most strongly enriched genes in rat PM, relative to all the other populations, were *Serpine1*, encoding plasminogen activator inhibitor-1 (PAI-1) and *Serpinb2*, encoding PAI-2. The selective and constitutive expression of SerpinB2 by mouse PM was reported previously (88). Rat PM also over-express multiple other serine protease inhibitors, *Slpi*, *Serpinb6*, *Serpinb9*, *Serpinb10* and *Serping1*, perhaps reflecting the large number of trypsin-binding proteins observed in peritoneal macrophage lysates (74). In addition to *Gata6*, the rat peritoneal macrophages selectively expressed multiple transcription factors, at least 2-fold higher than in AM, notably *Ahr*, *Mitf*, *Tfec*, *Batf3*, *Batf2*, *Stat1*, *Creb5*, *Mef2c*, *Id1*, *Etv1*, and *FoxP1*.

### The Impact of CSF1R Deletion in the Rat Liver

The recent development and characterisation of *Csf1r*^-/-^ rats permitted the analysis of brain (microglia) and spleen-specific macrophage gene expression profiles based upon the identification of transcripts that were selectively lost in the whole tissue gene expression profiles (27). In the case of microglia, the inferred profiles closely resembled the transcriptomes of microglia isolated from mice and humans. In the spleen, we identified a cluster of spleen-specific transcripts associated with the complete loss of the marginal zone macrophage populations in *Csf1r*^-/-^ rats.

Here we extended the comparative analysis to the liver, where we previously noted a substantial loss CD68^+^ macrophages by immunohistochemistry. The results are shown in **Table S4**. Consistent with possible functions of local growth factors in KC differentiation (89), genes encoding both CSF1R ligands (*Csf1* and *Il34*) were expressed in rat liver. Neither was affected by the *Csf1r* mutation. As we observed in other tissues, the expression of *Csf1r* mRNA in the heterozygous *Csf1r* rat livers was reduced by around 50% (i.e. there was no dosage compensation). There are substantial sex-specific fluctuations in gene expression profiles in the livers of rodents associated with periodic surges of growth hormone (90). We noted a set of transcripts including genes encoding the female-specific transcription factors *Cux2* and *Tsx1* and target genes (*A1bg*, *Ascl1*, *Lifr*, *Esr1*, *Prlr*) that were down-regulated in the female *Csf1r*^-/-^ livers and a reciprocal set including male-specific *Bcl6* and targets *Cyp2c11*, *Sult1e1*, and *Hsd3b5* that were highly up-regulated. These differences are likely related to the lack of ovarian development and female infertility in these rats (27).

We previously inferred the gene expression of profile of neonatal rat KC based upon the set of genes that was induced in the liver by treatment with CSF1 but was expressed at lower levels in BMDM (19). The coordinate loss of these transcripts is consistent with the reduction in liver macrophages by immunohistochemistry (27). We have subsequently demonstrated a complete lack of IBA1^+^ embryonic liver macrophages in *Csf1r*^-/-^ rats (91), which suggests the residual liver macrophages in these rats may be monocyte-derived and thus differ from embryo-derived KC. There was no apparent reduction in expression of either *Msr1* (the macrophage scavenger receptor) or *Lgals3* (Galectin 3) in *Csf1r*^-/-^ rat livers, which were shown to be expressed by IBA1^+^ rat KC (92). Aside from *Msr1* and *Lgals3*, many other known macrophage-associated transcripts were unaffected by *Csf1r* mutation, notably the lineage-specific transcription factors *Cebpb* and *Spi1*, *Mertk*, *Mrc1*, *Cd14*, the GM-CSF receptor (*Csf2ra*/*Csf2rbI*), *Clec7a*, *Tlr4*, *Gpnmb*, and *Ccr2*.

By contrast, transcripts encoding *Axl* (related to *Mertk*) and its major ligand, *Gas6*, were both highly-expressed in rat liver and reduced in *Csf1r* deficient rats. In mice *Axl* is protective against hepatoxic injury in response to LPS or CCl4 (93). Markers expressed by other cell types, *Pecam1* and *Cdh5* for endothelial cells (94), *Pdgfrb* (hepatic stellate cells), *Alb* (parenchymal cells), *Gls* and *Cyp2e1* (centrilobular cells) were unaffected by the loss of Csf1r-dependent macrophages. Transcription factors proposed as regulators of KC adaptation in mice include *Id3*, *Nr1h3*, *SpiC* and *Nfe2* (6, 95). Of these, *Id3* and *Nr1h3* were expressed at high levels but unaffected by *Csf1r* mutation and *SpiC* was barely detectable in rat liver. The evidence favoring a role for Id3 in KC adaptation was based in part upon conditional mutation using a *Tnfrsf11a*-cre driver (6) but *Tnfrsf11a* (encoding receptor activator of NF-kappaB, RANK) in mice is predominantly expressed in hepatocytes (96). Like *Id3*, it was unaffected by the *Csf1r* mutation in rat liver. Transcription factors that were apparently *Csf1r*-dependent in rats include *Nfe2*, *Hes6*, *Etv5, Creb3l1*, and *Tfec*. The latter is macrophage-specific in mice (97) and was also reduced in rat *Csf1r*^-/-^ spleens (27). However, the transcription factor that was most highly-expressed and *Csf1r-*dependent was the estrogen receptor, *Esr1*. Estradiol administration acts directly to modulate Kupffer cell function *via* Esr1 in both mice and rats (98, 99).

Overall, the data confirm that the rat shares elements of liver-specific macrophage adaptation with mouse and human and potentially reveal markers and transcriptional regulatory mechanisms specific to embryo-derived macrophages. We suggest that the residual CD68^+^ macrophages present in the *Csf1r*^-/-^ livers may be recently recruited monocyte-like cells that do not acquire the adapted transcriptome of KC.

## Conclusion

The availability of CRISPR-Cas9 technology and whole genome sequencing is rapidly transforming the practicality of the rat as an experimental animal that has many advantages over the mouse. Here we have described and validated a method for the generation of macrophages from rat ESC and provided a macrophage transcriptomic resource to enable comparative analyses of disease models in rats and mice.

## Data Availability Statement

The datasets presented in this study can be found in online repositories. The names of the repository/repositories and accession number(s) can be found in the article/**Supplementary Material**.

## Ethics Statement

The animal study was reviewed and approved by The University of Edinburgh.

## Author Contributions

CP, GD and LL performed experiments. CP, KI, SB and DH analysed array data. CP and DH conceived the idea for the study. CP, KI, and DH wrote the manuscript. DH secured funding for the study. All authors contributed to the article and approved the submitted version.

## Funding

This work was funded by the Medical Research Council UK (MR/M019969/1).

## Conflict of Interest

The authors declare that the research was conducted in the absence of any commercial or financial relationships that could be construed as a potential conflict of interest.
