# Supplementary material for: Transcriptomic Analysis of Rat Macrophages
Source: Front Immunol. 2021 Feb 1;11:594594. doi: 10.3389/fimmu.2020.594594 (PMC7902030; doi:10.3389/fimmu.2020.594594)
Supplement: Supplementary file 1 [file DataSheet_1.docx]

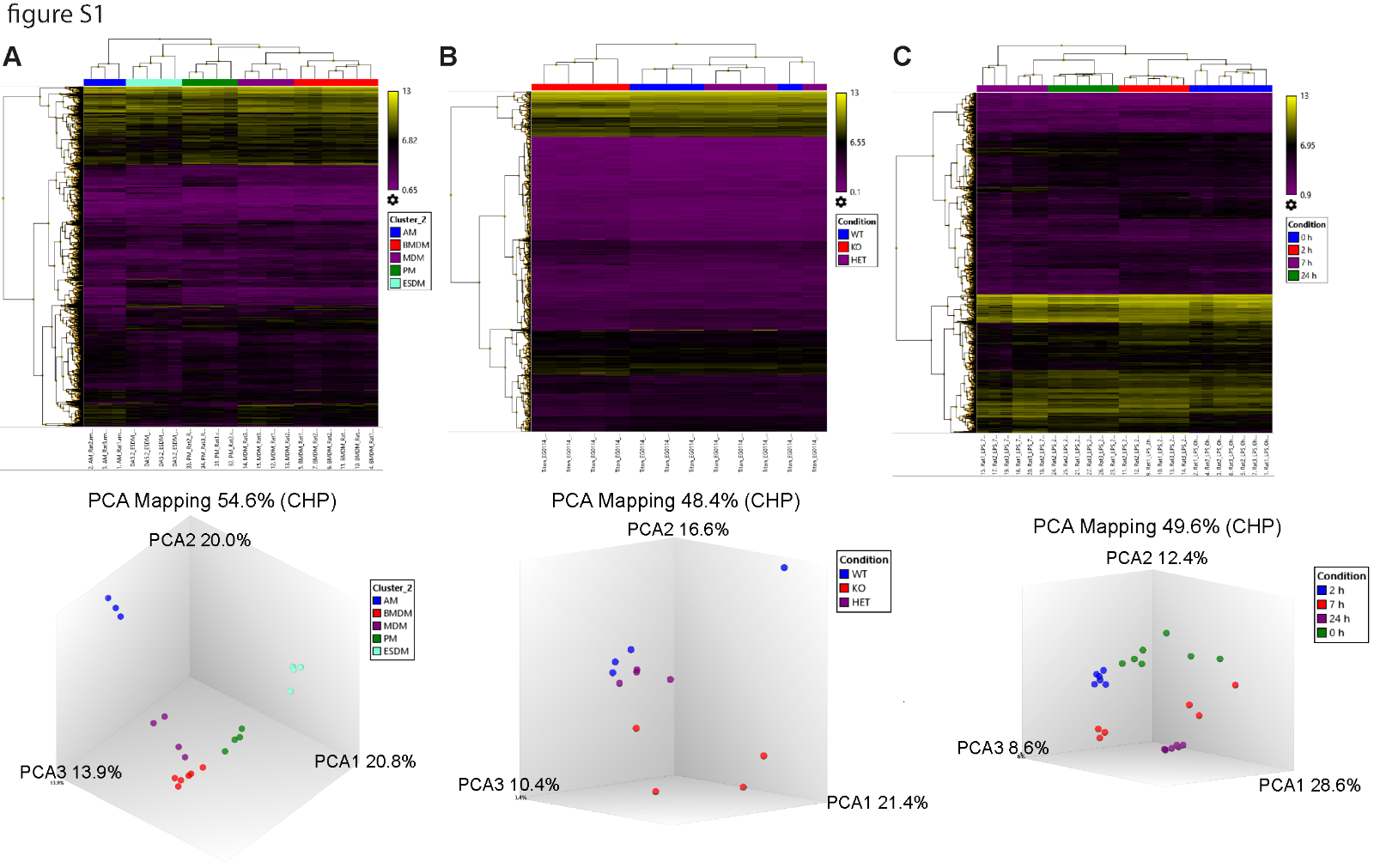

**Supplementary Figure 1 - Hierarchical clustering and PCA plots**

A) Rat Macrophages: Hierarchical clustering of the top 5000 differentially expressed genes sorted by false discovery rate (FDR; <0.009418). Three biological replicates were used for alveolar (AM), bone marrow-derived (BMDM), monocyte-derived (MDM) and peritoneal macrophages (PM), including technical replicates. Technical replicates were used ESC-derived macrophages (ESDM) obtained from a single ESC clone (DA5.2). B) Kupffer Cells: Hierarchical clustering of the 2652 differentially expressed genes sorted by FDR <0.05. Four biological replicates were used per genotype for *Csf1r*^+/+^ (WT), *Csf1r*^+/-^ (HET) and *Csf1r*^-/-^ (KO) rat livers. C) LPS time points: Hierarchical clustering of the top 5000 differentially expressed genes sorted by FDR <0.04298). Data was obtained from 3 adult wild-type rats on a DA background and included 2 technical replicates per rat. One array replicate was also included for 0 h LPS.


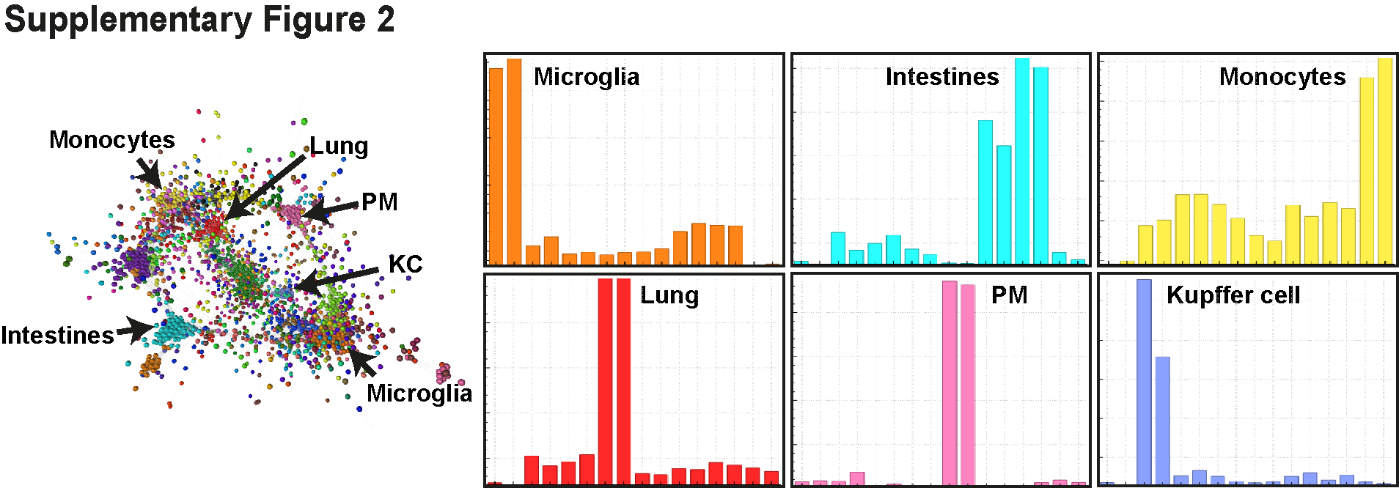


**Supplementary Figure 2 – Network analysis of Lavin data**

The differentially expressed genes identified by Lavin and colleagues (Lavin et al., 2014) were clustered using the network analysis tool Graphia (Freeman et al., 2020). A Pearson correlation of R = 0.85 and MCL clustering with an inflation value of 1.8 was used to identify alveolar macrophage (lung), peritoneal macrophage (PM) and Kupffer cell gene signatures. The figure shows the network graph generated by the analysis in which genes are coloured by clusters of co-expression. Histograms show expression profiles of clusters that contained genes specific to each macrophage population.





**Supplementary Figure 3 – Analysis of mouse alveolar macrophage gene expression in rat**

The expression of genes in the mouse alveolar macrophage (AM) cluster is shown on the left (using data from (Lavin et al., 2014)). The graph on the right shows the expression of these genes in rat AM. The genes represented by open bars were common to both mouse and rat AM clusters. Rat genes denoted with an asterisk (*) have provisional or model RefSeq status on the Rat Genome Database (rgd.mcw.edu). Graphs show mean + SEM.

**

**

**Supplementary Figure 4 – Analysis of mouse peritoneal macrophage gene expression in rat**

The expression of genes in the mouse peritoneal macrophage (PM) cluster is shown on the left (using data from (Lavin et al., 2014)). The graph on the right shows the expression of these genes in rat PM. The genes represented by open bars were common to both mouse and rat PM clusters. Rat genes denoted with an asterisk (*) have provisional or model RefSeq status on the Rat Genome Database (rgd.mcw.edu). Graphs show mean + SEM.





**Supplementary Figure 5 – Analysis of mouse KC gene expression in wild-type and *Csf1r*^-/-^ rat livers**

The expression of genes in the mouse Kupffer cell (KC) cluster is shown on the left (using data from (Lavin et al., 2014)). The graph on the right shows the expression of these genes in wild-type (+/+) and
*Csf1r*^-/-^ (-/-) rat livers. The genes represented by open bars or within the dotted box were common to both mouse and rat KC clusters. Rat genes denoted with an asterisk (*) have provisional or model RefSeq status on the Rat Genome Database (rgd.mcw.edu). Graphs show mean + SEM. p = 0.0007 (*** *Clec4f*), 0.0414 (* *Folr2*), 0.0024 (** *Vsig4*), 0.0169 (* *Gpr182*), 0.0193 (* *Ehd3*), 0.0298 (* *Gnmt*) and 0.0209 (* *Hpd*) via an unpaired t-test.

**References**

Freeman, T.C., Horsewell, S., Patir, A., Harling-Lee, J., Regan, T., Shih, B.B., et al. (2020). Graphia: A platform for the graph-based visualisation and analysis of complex data. *bioRxiv*. doi: <https://doi.org/10.1101/2020.09.02.279349>

Lavin, Y., Winter, D., Blecher-Gonen, R., David, E., Keren-Shaul, H., Merad, M., et al. (2014). Tissue-resident macrophage enhancer landscapes are shaped by the local microenvironment. *Cell* 159**,** 1312-1326. doi: 10.1016/j.cell.2014.11.018
